# AnatomySketch: An Extensible Open-Source Software Platform for Medical Image Analysis Algorithm Development

**DOI:** 10.1007/s10278-022-00660-5

**Published:** 2022-06-29

**Authors:** Mingrui Zhuang, Zhonghua Chen, Hongkai Wang, Hong Tang, Jiang He, Bobo Qin, Yuxin Yang, Xiaoxian Jin, Mengzhu Yu, Baitao Jin, Taijing Li, Lauri Kettunen

**Affiliations:** 1 School of Biomedical Engineering, Faculty of Electronic Information and Electrical Engineering, Dalian University of Technology, Dalian, 116024 China; 2 Faculty of Information Technology, University of Jyväskylä, 40100 Jyväskylä, Finland; 3 Liaoning Key Laboratory of Integrated Circuit and Biomedical Electronic System, Dalian, 116024 China

**Keywords:** Medical image analysis, Image annotation, User interaction, Algorithm development, Deep learning

## Abstract

The development of medical image analysis algorithm is a complex process including the multiple sub-steps of model training, data visualization, human–computer interaction and graphical user interface (GUI) construction. To accelerate the development process, algorithm developers need a software tool to assist with all the sub-steps so that they can focus on the core function implementation. Especially, for the development of deep learning (DL) algorithms, a software tool supporting training data annotation and GUI construction is highly desired. In this work, we constructed AnatomySketch, an extensible open-source software platform with a friendly GUI and a flexible plugin interface for integrating user-developed algorithm modules. Through the plugin interface, algorithm developers can quickly create a GUI-based software prototype for clinical validation. AnatomySketch supports image annotation using the stylus and multi-touch screen. It also provides efficient tools to facilitate the collaboration between human experts and artificial intelligent (AI) algorithms. We demonstrate four exemplar applications including customized MRI image diagnosis, interactive lung lobe segmentation, human-AI collaborated spine disc segmentation and Annotation-by-iterative-Deep-Learning (AID) for DL model training. Using AnatomySketch, the gap between laboratory prototyping and clinical testing is bridged and the development of MIA algorithms is accelerated. The software is opened at https://github.com/DlutMedimgGroup/AnatomySketch-Software.

**Supplementary Information:**

The online version contains supplementary material available at 10.1007/s10278-022-00660-5.

## Background

Nowadays, computer-assisted medical image analysis (MIA) algorithms are increasingly used in disease diagnosis and treatment. The development of MIA algorithms is a complex process involving algorithm design, model training, software implementation and performance testing. To speed up the development, researchers need a convenient software platform to assist with different sub-step of the process. Ideally, the platform should include a graphical user interface (GUI) for user interaction and data visualization, as well as a plugin interface for user-developed algorithm integration. Many software tools have been established to meet these needs. Several code libraries were developed to help with algorithm implementation, such as the classical itk [1, 2], vtk [3], elastix [4, 5], ANTS [6] and the recently published libraries for radiomics (e.g. pyradiaomics [7]) and deep learning (e.g. monai^1^). GUI-based software tools were also developed for image segmentation (e.g. ITK-SNAP [8], MITK [9], TurgleSeg [10], Seg3D), data annotation (DicomAnnotator [11] and Pair^2^) and the analysis of specific imaging modalities (e.g. SpheroidJ [12], MNI SISCOM [13] and OIPAV [14]). Few of these tools are extensible for user-developed algorithms including deep neural networks. The 3D Slicer [15] software has powerful extension capabilities and a rich library of extension modules [16, 17], but its programming mechanism and interaction workflow are relatively complex for junior programmers.

In recent years, deep learning (DL) algorithms are increasingly used in clinical applications. To alleviate the heavy burden of data annotation for DL model training, the procedure of Annotation-by-iterative-Deep-Learning (AID) becomes popular. In the AID workflow, the human experts first annotate a small set of training data which are used for training a preliminary annotation network. The preliminary network is then used to automatically annotate more training data with imperfect accuracy. Next, the human expert proofread the network-annotated data to ensure the annotation accuracy, and the proofread data are supplemented to the training set to retrain a more accurate annotation network. As this process is repeated, the network becomes more and more accurate; thus, fewer and fewer human effort is needed for data annotation. To support the AID workflow, a software platform with convenient annotation/proofreading tools and a plugin interface for the annotation network model is necessary. Philbrick et al. developed the RIL-Contour [18] software with AID function which supports multiuser collaboration and network model version management. However, this software only focuses on data annotation; it is not a general assistance tool for the entire algorithm development workflow.

Besides the needs of a software assisting the classical MIA algorithm development, the recently trend of DL algorithm research also requires a software platform to integrate neural network models and the AID workflow. Due to the lack of such a platform, researchers tediously switch between different assistance tools, making the development process complicated and slow. Moreover, without a GUI-based platform, many algorithms are published as command-line tools or even source codes that are unfriendly to clinical users.

In response to the existing needs, we developed a software platform named AnatomySketch for fast MIA algorithm integration and GUI-based software prototyping. AnatomySketch (AS) offers convenient tools for data annotation, image visualization and algorithm integration, so that the algorithm developers can focus on core function development and rapidly produce a software prototype for algorithm demonstration and testing. Compared to the existing software tools, AnatomySketch has an easier interface for DL model integration and more convenient supports for multi-touch and stylus-based image annotation. The software is developed with the following key features:

(i) *A convenient GUI for data visualization and human–computer interaction.* AnatomySketch has a concise interface for multi-modality and multi-dimensional image visualization. It also incorporates a library of basic processing tools for medical images and graphical models to save the time of basic processing function implementation. To support the development of semi-automated algorithms and the annotation of training data, AnatomySketch provides simple workflow for data annotation mouse, shortcut keys, stylus and multi-touch screen. It also facilitates a simple correction of the automatic segmentation with an inbuilt contour editing method.
(ii) *Flexible plugin interface for user-developed algorithms.* With a flexible plugin interface, the software allows the users to integrate their algorithms (including DL models) as extension function modules. This feature facilitates rapid prototyping of GUI-based software for specific clinical applications, making the evaluation and demonstration of novel algorithms easier and faster. By combining the DL model plugins with the annotation tools, the AID workflow can be realized to speed up DL model training.

The following sections will introduce the detailed software feature and demonstrate exemplar applications of fast software prototyping and AID workflow realization.

## Method

### Software Design and Architecture

AnatomySketch is designed with a concise GUI consisting of a menu bar (on the top), a data list for data property management (on the top-left), a customizable widget panel for user-developed function modules (on the bottom-left) and a display area (on the right) with three orthogonal section windows and one three-dimensional (3D) view window. The design philosophy of the GUI is being simple, intuitive and familiar to the MIA researchers. The GUI has two operation modes, namely the desktop mode for mouse and keyboard interaction and the tablet mode for stylus and touch screen interaction.

**Fig. 1:**
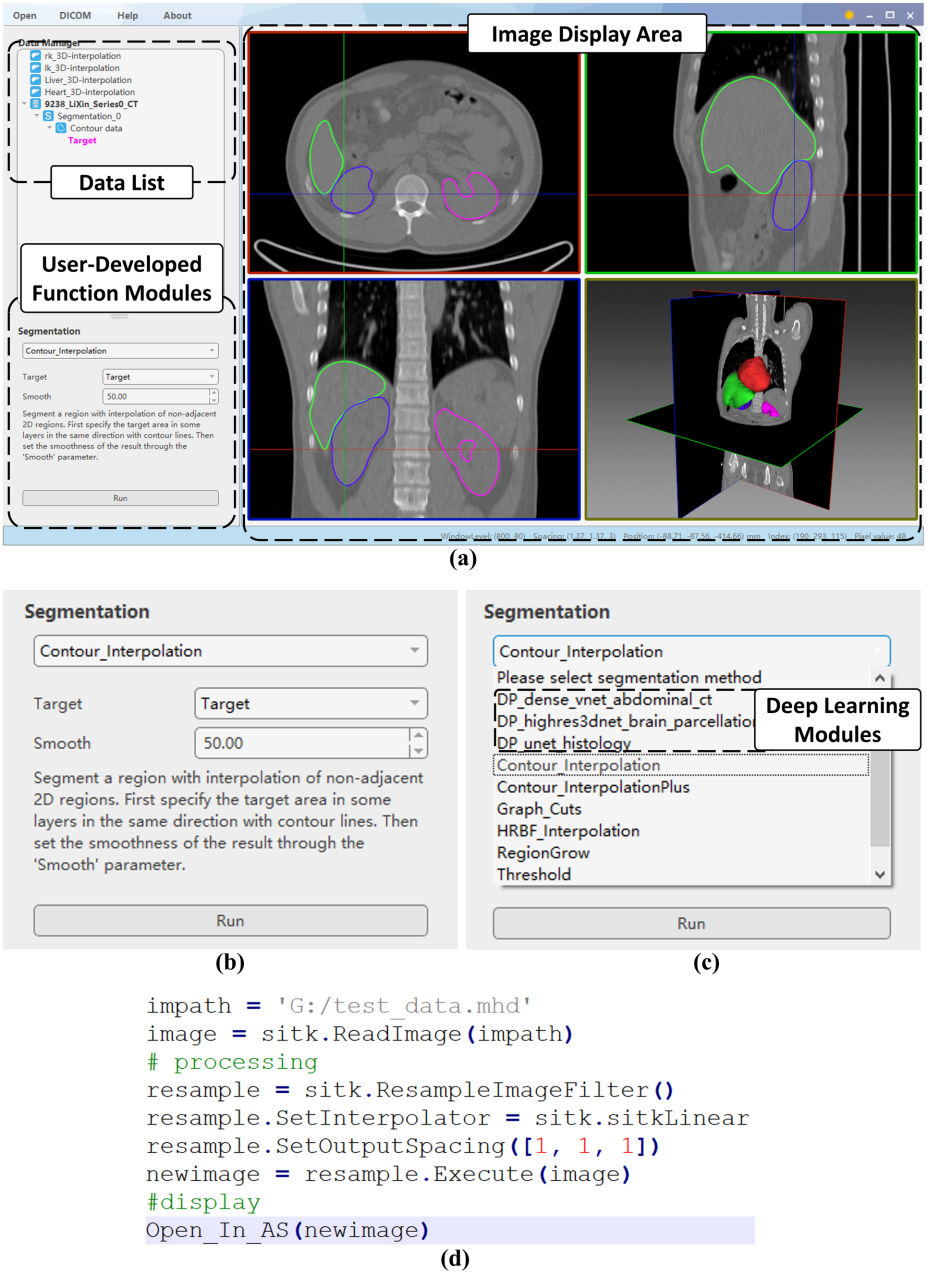
**a** AnatomySketch interface. **b** The function module panel. **c** An example of the drop list of user-defined function modules, mostly segmentation methods in this case, including deep network models. **d **An example of calling the software GUI (the highlighted line of code) to visualize intermediate variables.

The desktop mode has a classical layout (Fig. 1a) similar to the well-known MITK, ITK-SNAP and 3D Slicer. Distinctively, the layout has a customizable widget panel for user-developed algorithms on the bottom-left (Fig. 1b). A drop list (Fig. 1c) on top of the panel allows the selection of algorithm modules, and the panel layout changes according to the selected algorithm. The algorithm-specific panel layout is defined via a configuration file specifying the positions and appearances of the control widgets (including drop menu, text box, press button and explanatory text). Details of the layout definition will be introduced in the “Extension Module” section.

To assist with data visualization during algorithm debugging, we also allow the desktop mode GUI to be called as an inline function of the user programs (e.g. Python, MATLAB or C +  + code). This feature is especially useful for accepting user interaction during the algorithm workflow or for inspecting the intermediate variables of the data flow. The intermediate variables can be images, graphical shape models and user-annotations. It is possible to overlay multiple variables in the display window to check the accuracy of image segmentation and/or registration. Figure 1d shows a line of Python code (namely the “Open_In_AS” function) calling the GUI for intermediate image inspection. This Python code is available on the website of AnatomySketch.

The tablet mode is developed to take advantage of the multi-touch screen and the stylus (if available) for efficient annotation of region boundaries. This mode can be activated by clicking expansion button on the top-right of the display windows. The clicked window is enlarged as a palette for stylus sketching. As shown in Fig. 3a, the operator can use the stylus with one hand to drawn contours and scribbles, and meanwhile use the other hand to zoom, pan or rotate the image via the multi-touch screen.

**Fig. 2:**
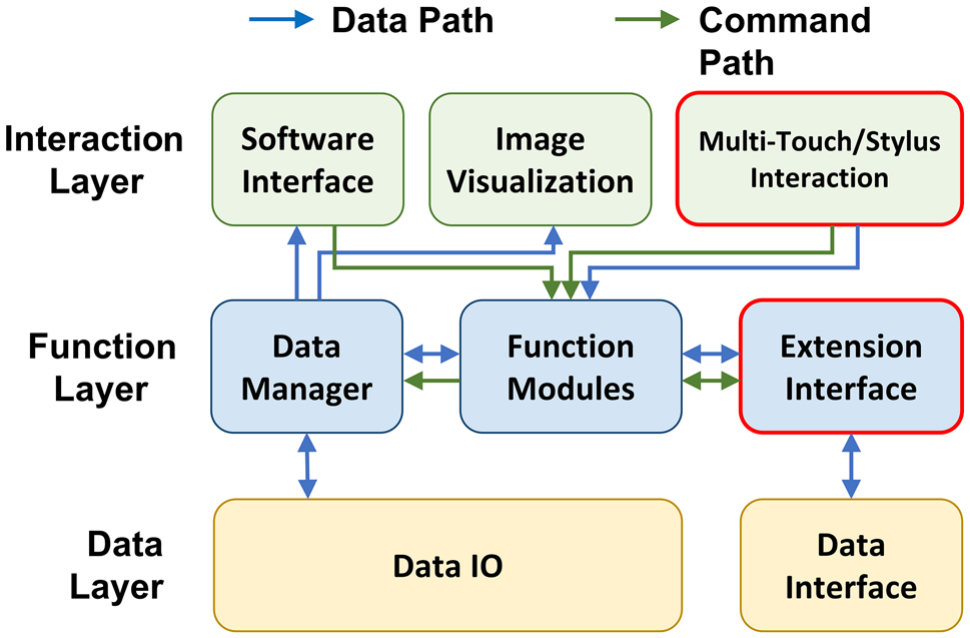
The architecture diagram of the software platform

The architecture of the AS software is shown in Fig. 2. The software architecture is composed of three layers including the interaction layer, the function layer and the data layer. The blue and green arrows denote the data and command paths between the modules, respectively. As the core of the software, the function modules coordinate all the other modules to handle data processing and user interactions.

### Interactive Annotation and Proofreading

**Fig. 3:**
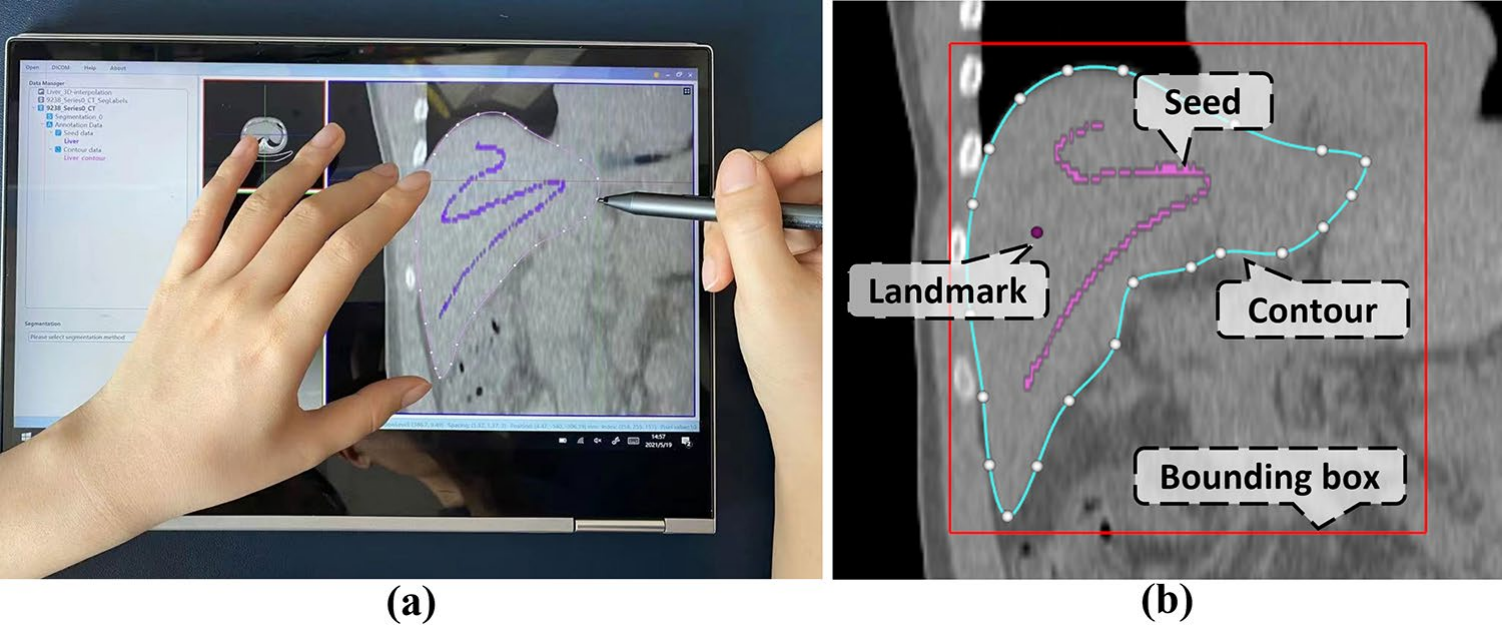
Interactive data annotation. **a** The tablet mode layout supporting stylus sketching and multi-touch gestures. **b** Multiple annotation tools are provided by the software

As shown in Fig. 3b, AnatomySketch supports the annotation of anatomical landmarks, bounding boxes, edge contours, curves, scribbles and object regions using the mouse or the stylus. All the annotations can be accessed by the user-developed plugin modules as inputs. The annotations can also be exported into computer discs as separate files for offline algorithm training.

**Fig. 4:**
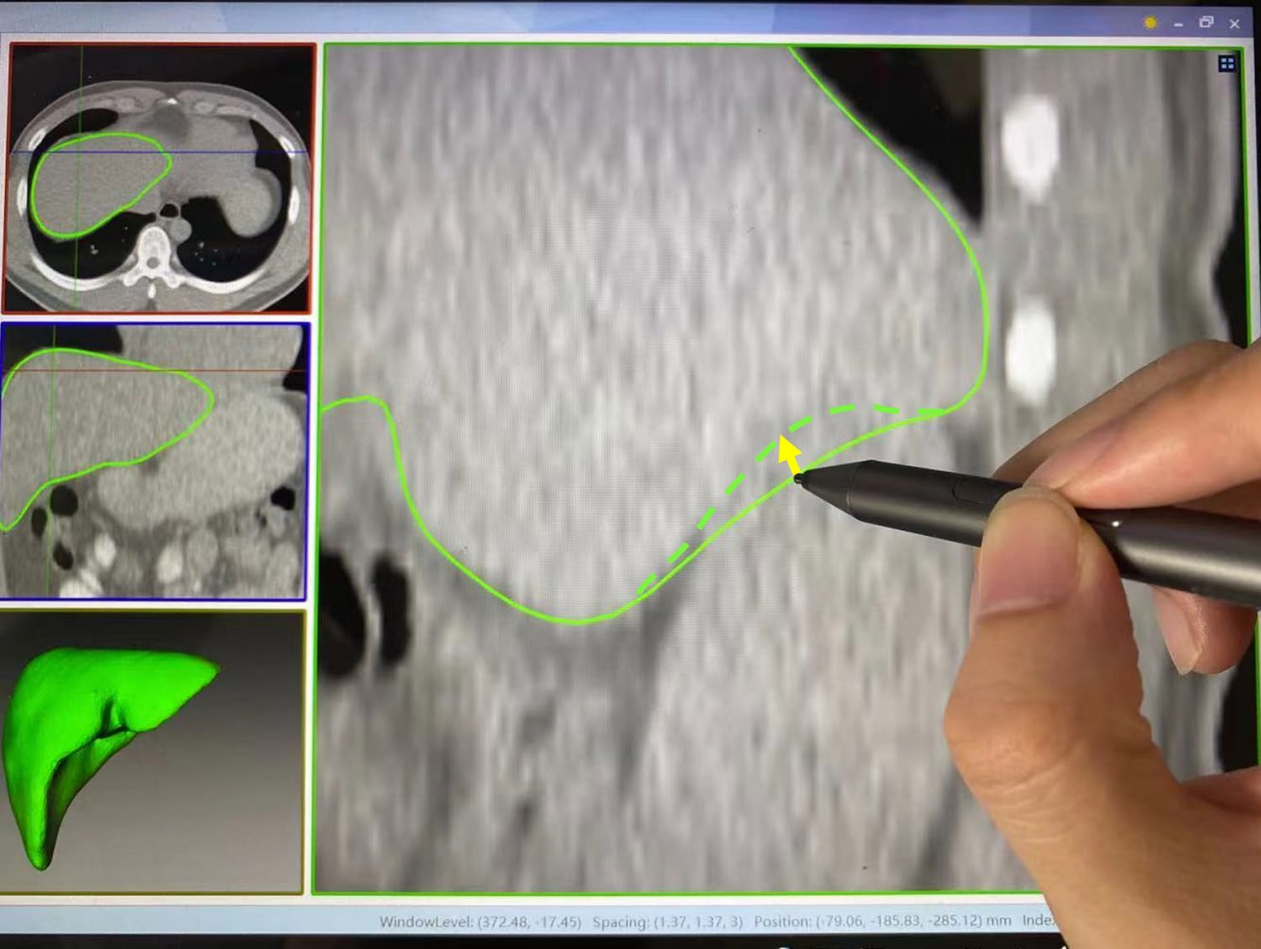
Proofreading of the automatic segmentation result. The inaccurate boundary can be dragged towards the correct position (the dashed curve) using the stylus or the mouse

AnatomySketch also provides a convenient boundary correction tool for proofreading the segmentation results of automatic algorithms. This tool is implemented based on the free-form deformation (FFD) method [19]. It allows the user to drag the inaccurate boundary towards the correct position (as shown in Fig. 4). We applied both 2D and 3D versions of the FFD method for adjusting 2D contours and 3D surfaces, respectively.

FFD is a point-controlled contour deformation method. After the user dragging operation, a $6\times 6$ grid is constructed around the starting point of the dragging. The mouse/stylus motion vector (yellow arrow in Fig. 4) is first extrapolated to the $6\times 6$ grid vertices by solving an inverse interpolation function of the cubic B-spline. Then, the deformation vectors of contour points are interpolated by solving the cubic B-sample interpolation function,

1 $$\begin{array}{c}{\text{p}}=\sum\limits_{i=0}^{3}\left(\begin{array}{c}3\\ i\end{array}\right){\left(1-m\right)}^{3-i}{m}^{i}\left(\sum\limits_{j=0}^{3}\left(\begin{array}{c}3\\ j\end{array}\right) {\left(1-n\right)}^{3-j}{n}^{j}{\text{p}}_{i,j}\right)\end{array}$$

where ${\text{p}}$ is the interpolated deformation vector of a contour vertex, $(m,n)$ is the normalized local coordinate of the dragging start point, and ${\text{p}}_{i,j}$ is the deformation vector of grid node $(i,j)$. The readers are referred to [19] for more details of the method.

### Extension Module

**Fig. 5:**
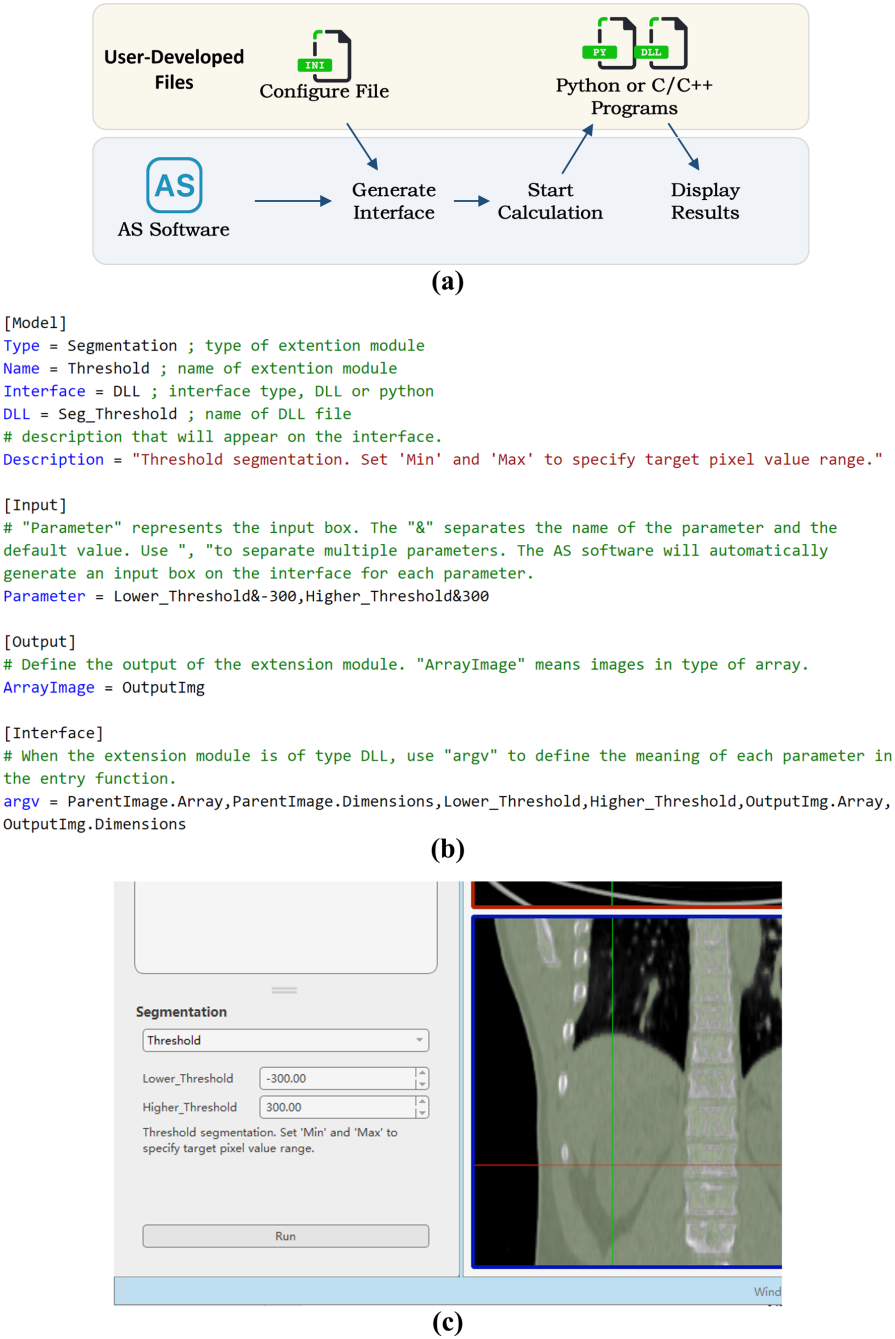
The extension module. **a** The workflow of the extension module. **b** An example of the configuration file. **c** The widget panel generated by AnatomySketch according to the configuration file of (**b**)

AnatomySketch provides a flexible plugin interface for the integration of user-developed algorithms. Figure 5a shows the workflow of the extension modules. A configuration file and a program file simply form the plugin interface of the software. The configuration file is a text-format definition file specifying the extension module type, input and output parameters and the GUI design. The software will read this definition file and realize the extension interface accordingly. The program file is the user-programmed executable (exe) file or a dynamic link library (dll) of the core algorithm function. Figure 5b shows an example of the configuration file for thresholding segmentation. The configuration file is imported into the software to create the customized widget panel (Fig. 5c). The program file is invoked by clicking the “Calculate” button on the widget panel. To transfer input and output data between the software and the program file, a loose coupling mechanism is adopted. The software first writes the input data (i.e. image arrays, polygonal meshes or annotations) into the computer disc, then the user program imports them for computation and writes the outputs to the computer disc. Finally, the software gets the output results from the disc and updates the GUI display.

On the AnatomySketch website, several quick-start templates of the configuration files and program files are provided. The developers can publish their algorithm modules on the website to promote the usage of their works.

### Deep Learning Support

Thanks to the flexible plugin interface, deep neural networks can be integrated into the software as plugin modules. The developer needs to create a Python-based program file to get input data from the software and calls the network model to process the data. The network can be developed using any DL platform (e.g. PyTorch, TensorFlow or Keras) and compiled as an executable file to be called by the user-created Python program. The outputs of the network are written into the hard drive and then imported into the software via the plugin interface.

In AnatomySketch, the AID workflow is realized by combining the annotation tools, the proofreading tools and the plugin modules. Rich annotation tools of AnatomySketch are used for labelling the primary training data, then a user-defined Python program is called to train the network and use the trained model to label more images. During the proofreading, the 2D and 3D FFD tools are used to correct the segmentation errors. The entire AID workflow is customized as a plugin module with a widget panel supporting iterative training and proofreading. Video 1 attached to this paper demonstrates the AID support features of AnatomySketch.

## Results

In this section, we will demonstrate the examples of using AnatomySketch for fast plugin module development and software prototyping. We will also show examples of human-AI collaborated image segmentation and AID-based data annotation. These examples demonstrate the convenience of integrating DL models or interactive algorithms into our software, while such integration can be time consuming or even infeasible for the existing tools.

### Software Prototyping

**Fig. 6:**
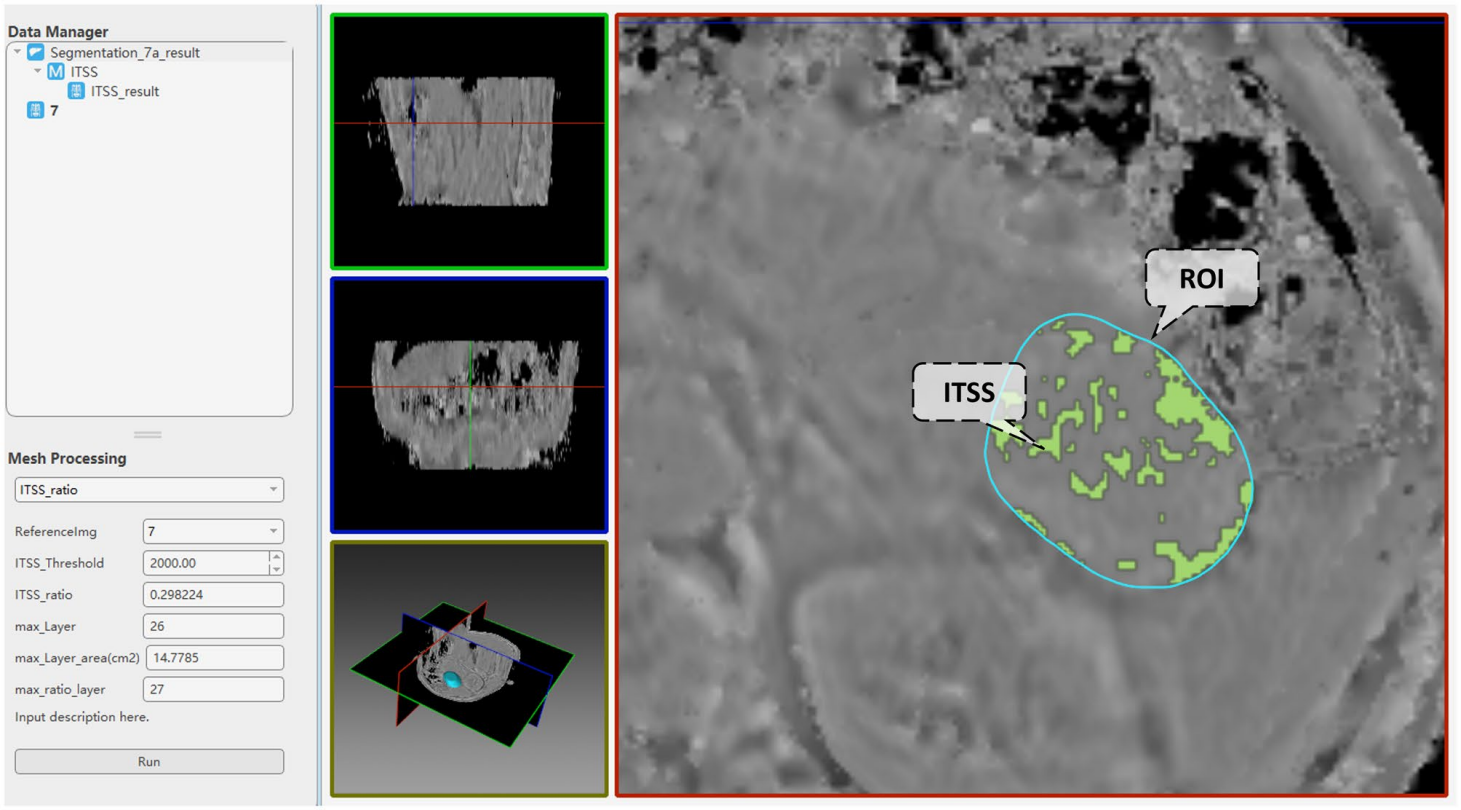
An example of user-defined extension module for ITSS analysis in MR images

In the first example, AnatomySketch was used in a medical research project for analyzing intratumoral susceptibility signal intensities (ITSS) in enhanced T2 angiography of hepatocellular carcinoma. Because this study uses special MRI pulse sequences for ITSS imaging, customized software needs to be developed for the data analysis. As a collaborator of this study, our group took only 1 day to develop a plugin module and created a GUI-based software prototype for the ITSS MR image analysis. With our software prototype, the doctors contoured the region of interest (ROI) in a few interleaved axial slices and interpolated the 3D surface from the 2D contours. The proofreading tool was used to adjust the 3D surface to precisely fit the tumour boundary. The software automatically removed the MR imaging artefact and extracted the high-ITSS voxels via thresholding. The algorithm parameters such as ITSS threshold can be adjusted in the customized widget panel. Figure 6 shows the software GUI. The blue contour represents the ROI boundary and the green pixels represent extracted high-ITSS voxels. This tool has been used in a series of published studies [20–23]. Although the development of this module is simple and straightforward in the AS platform, similar extension may require time-consuming software recompilation in some existing tools (e.g. for MITK and ITKsnap).

**Fig.7:**
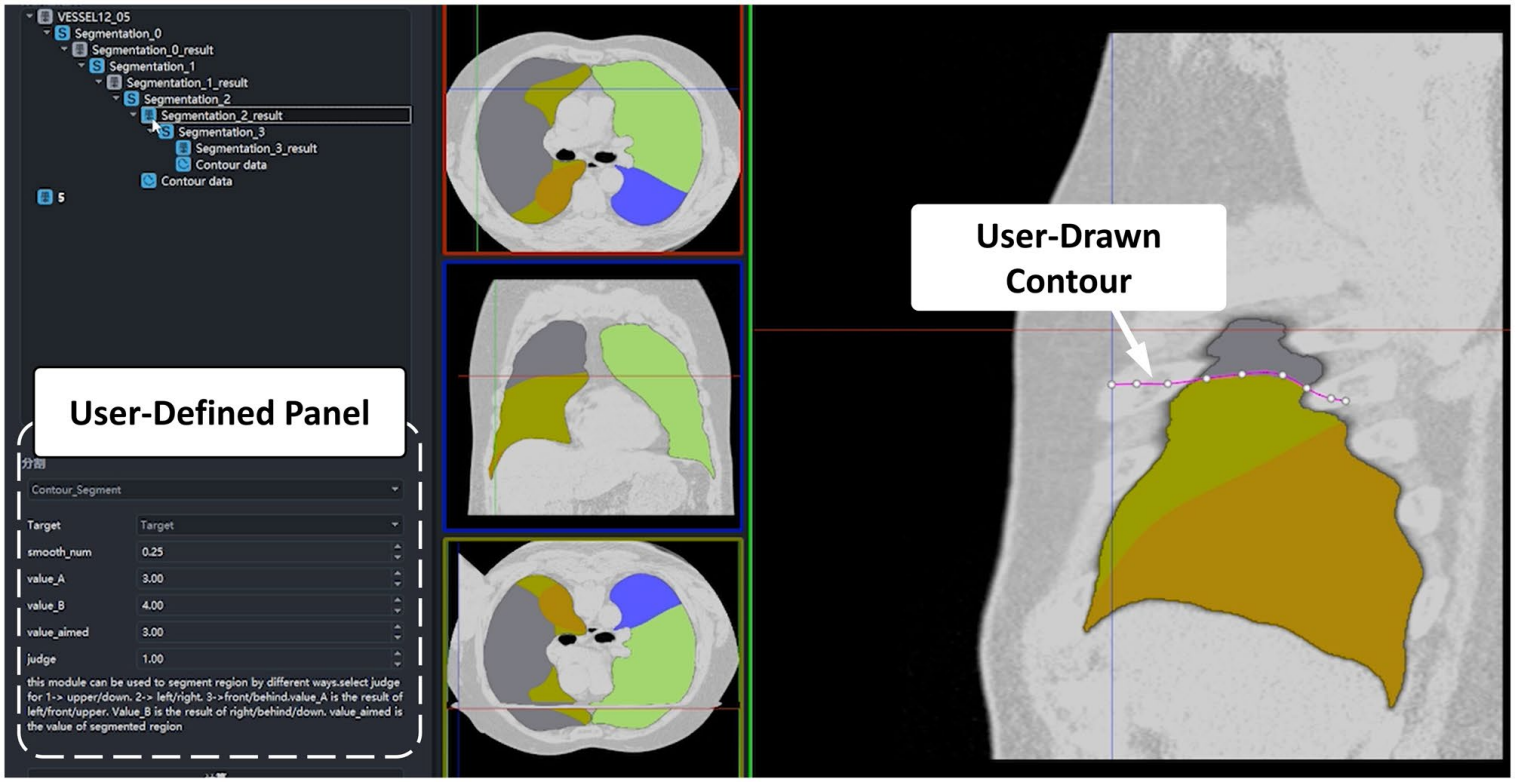
An example of user-developed plugin module for lung lobe annotation in CT images

In another example, AnatomySketch was used to annotate lung lobes in CT images for training a lobe segmentation network of commercial software. Engineers from a local company created an interactive lung lobe annotation plugin for AnatomySketch. The contours of lung fissures are manually sketched in a few coronal slices and a complete fissure surface was interpolated from the contours using radial basis function (RBF) interpolation. The proofreading tools of AnatomySketch were used to adjust the interpolated fissure surfaces. Figure 7 shows the GUI of the plugin module; VIDEO 2 attached to this paper exhibits the working process of this plugin module. Using this module, the annotation of five lung lobes took less than 20 min per image. Two engineers from the company annotated 100 CT images in 3 days, thanks to the stylus support of our software. In contrast, stylus interaction is not specially optimized in any other existing tool.

### Deep Learning Supports

**Fig. 8:**
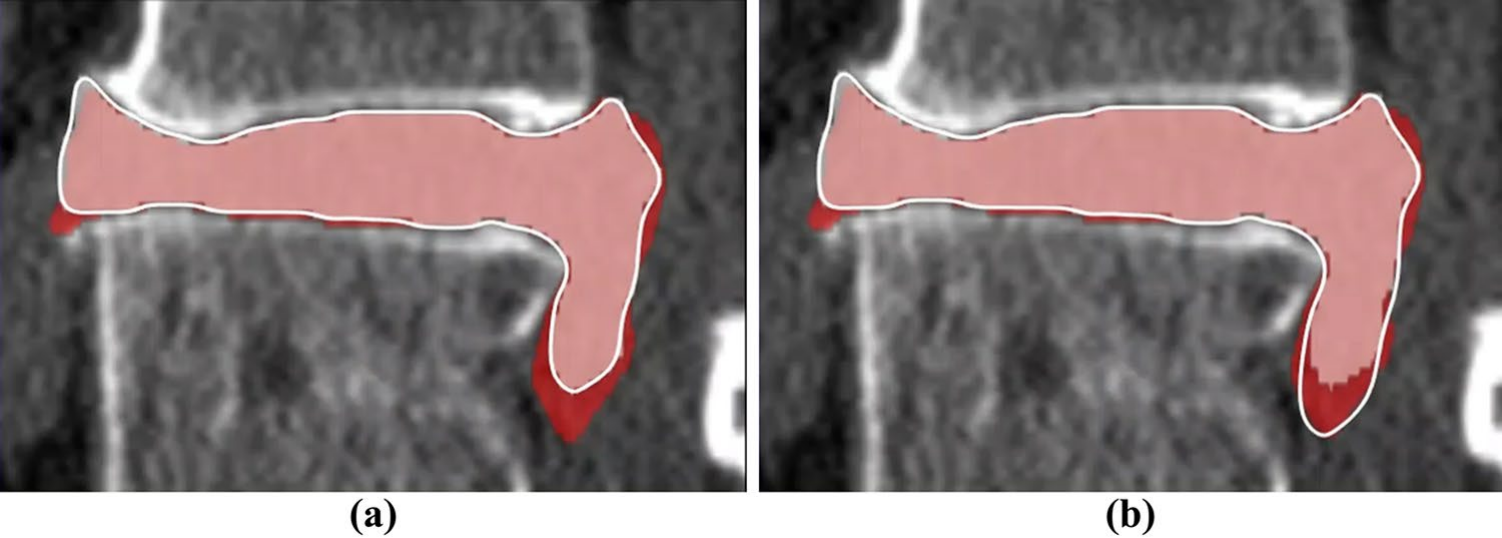
An example of 3D FFD proofreading. **a** The pink area in white contour is the automatic segmentation result of V-Net model. The red area and the white contour depict the under-segmented part. **b** Human expert proofreading result (the adjusted white contour) using the FFD tool

Through the plugin interface, user-developed deep network models are integrated into AnatomySketch as extension modules. As shown in the example of Fig. 8, a dense V-Net model [24] was trained to segment the intervertebral disc and the surrounding nerves and vessels from lumbar CT images. Due to the lack of enough training data, the network occasionally produced inaccurate segmentation at the fuzzy boundary of the herniated discs (Fig. 8a). Thanks to the 3D FFD proofreading tool, a human expert was able to correct the inaccurate segmentation within 5 min per image (Fig. 8b). In this way, the AI model and human expert collaborate with each other to achieve efficient and accurate segmentation of anatomical objects with weak boundaries. As a comparison, none of the existing medical image processing tools facilitate such efficient and direct proofreading of the DL segmentation results. Both MITK and 3D Slicer provide the AI-assisted annotation plugins, but they require Internet connection to the NVIDIA AI-Assisted Annotation Server for data transfer, which is inconvenient for the applications without Internet connections or with data privacy concerns.

**Fig. 9:**
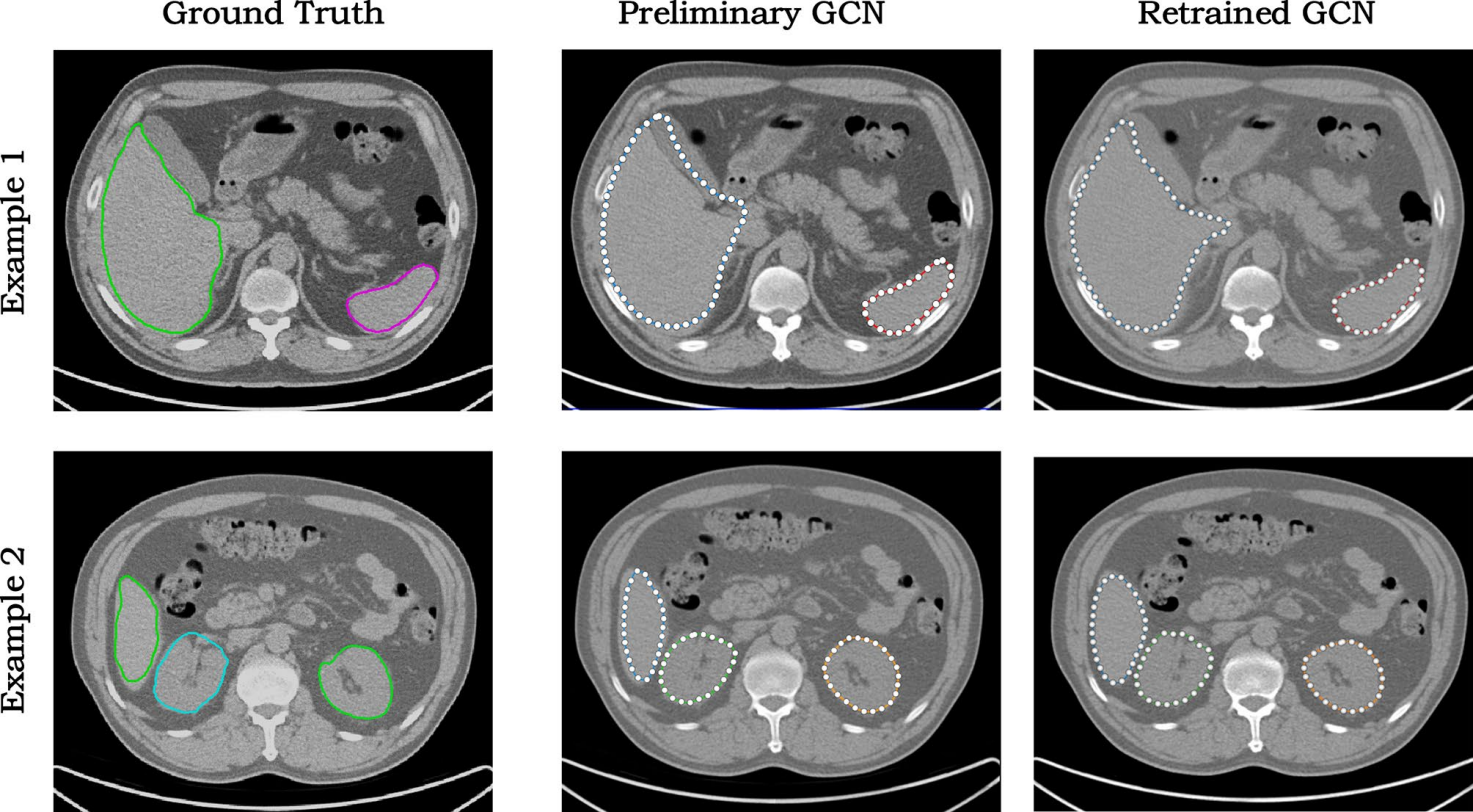
AID annotation results of two exemplar CT slices, showing that the retrained network yield more accurate results than the preliminary network. The ground truth comes from human expert manual labelling

Another example of human-AI interaction is the realization of AID workflow. We trained a DeepSnake network [25] to segment abdominal organs from CT images. The network generates 2D contours surrounding the target organs and deforms the contours to fit the organ boundaries. We first used a small set of expert-labelled training images to train a preliminary network, then used the preliminary network to generate the organ contours of more images. The automatically generated contours are proofread by human experts using the 2D FFD tool of AnatomySketch, and the images with proofread contours are supplemented to the training set to finetune the network. Figure 9 displays the predicted contours of the preliminary and retrained models for two representative slices, respectively. It is obvious that the retrained network (trained with about 1800 slices of 15 CT series) yields more accurate contour prediction than the preliminary network (trained using about 600 slices of five CT series).

## Discussions

AnatomySketch is developed with the goal of accelerating MIA algorithm development and bridging the gap between laboratory research and clinical application. The software is designed to meet many specific needs of MIA algorithm development:

(i) *Software prototyping.* During the past decade, many MIA algorithms were developed as command-line tools due to the difficulty of GUI construction. This problem also hampers the promotion of DL-based algorithms. Many DL models are published as source codes that cannot be tested by clinical users. AnatomySketch tackles this problem with a flexible plugin interface. As demonstrated in the examples of Figs. 6 and 8, the prototyping of a new software tool for special image modality (e.g. the ITSS MR) or specific application task (e.g. the segmentation of lumbar disc herniation) took less than 1 day. With a friendly GUI, the promotion of new MIA algorithms in the clinical environment becomes faster and more convenient.
(ii) *Data annotation.* The fast popularization of deep learning techniques proposes strong needs for data annotation. AnatomySketch provides annotation tools and the tablet mode for efficient image annotation. It also realizes the AID workflow for iterative annotation and training. An AID workflow is developed for abdominal organ segmentation based on the DeepSnake network. Thanks to the flexible plugin interface, AnatomySketch is versatile enough for realizing task-specific AID workflow based on different network models.
(iii) *Human–computer interaction.* Because fully automatic algorithms may not guarantee robustness in complex clinical scenario, semi-automatic algorithms with human guidance and corrections become practical choices. As shown in the example of Fig. 7, the plugin module interpolates the lung fissure surfaces using human-annotated curves as the guidance. Figure 9 demonstrates another example in which human interaction is introduced to correct the DL model output. AnatomySketch supports both input guidance and posterior correction to the DL models, facilitating flexible human-AI collaboration for clinical image analysis.

When comparing AnatomySketch with existing MIA software tools, we find that some key features of AnatomySketch have been integrated into the existing tools. The extension module is also available in the Slicer software [15, 16]. The integration of DL models has been realized by the MITK NVIDIA clara plugin^3^ and the RIL-contour software [18]. The AID workflow is also supported by the RIL-contour software. However, these software tools were only designed to assist with a certain step of the entire algorithm development workflow. The advantage of AnatomySketch is the supporting of the complete workflow, including data visualization, image annotation, algorithm integration and software prototyping.

As a newly established software, the number of user-developed plugin modules for AnatomySketch is still growing, especially for specific clinical applications. We will keep maintaining the web community to help the developers sharing their plugin modules and gain potential users from universities and hospitals. We also plan to add online crowdsourcing tools for multi-rater annotation and proofreading. Moreover, because AnatomySketch is increasingly used by the doctors who do not share the medical images, we will incorporate federated learning [26] ability for multi-centre model training without sharing confidential medical data. A plugin module will be developed to allow AS software to communicate with multi-centre client databases and to invoke models for inference. In this scenario, physicians at each centre can use the tools provided in AS to annotate images and invoke network models with AS interface.

## Conclusion

We developed a medical image analysis software platform named AnatomySketch to assist with MIA algorithm development. The software is specially designed for efficient image annotation and convenient integration of user-developed algorithm modules including deep neural networks. The AID workflow can also be realized to accelerate the training of DL models. For the next step, we will construct a web community for sharing user-developed extension modules and incorporate federated learning to facilitate mutual learning between DL models from multi-centres.

## Supplementary Information

Below is the link to the electronic supplementary material.

Supplementary file1 (MP4 67907 KB)

Supplementary file2 (MP4 1013 KB)

## Data Availability

The Declarations

## Footnotes

1 Monai: https://monai.io/

2 Pair: http://www.aipair.com.cn/

3 MITK NVIDIA clara plugin: https://www.mitk.org/wiki/MITK_ReleaseNotes_2018.04.2

## Code Availability

The Anatomy Sketch software is opened at https://github.com/DlutMedimgGroup/AnatomySketch-Software.
